# Process Integration and Life‐Cycle Assessment of Moist‐Solid Hydrolysis of Polylactic Acid with Lactic Acid Recovery via Electrodialysis

**DOI:** 10.1002/cssc.202500503

**Published:** 2025-05-20

**Authors:** Hui Luo, Dingchang Yang, Jhuma Sadhukhan, Verdeluz Costica, Robert Dorey, Qilei Song, Maria‐Magdalena Titirici

**Affiliations:** ^1^ School of Engineering University of Surrey Stag Hill Campus Guildford GU2 7XH UK; ^2^ Department of Chemical Engineering Imperial College London South Kensington Campus London SW7 2AZ UK

**Keywords:** chemical recyclings, electrodialyses, hydrolyses, life‐cycle assessments, polylactic acids

## Abstract

Poly(lactic acid) (PLA), a biodegradable plastic derived from starch, has gained prominence as a sustainable alternative to petroleum‐based plastics. However, the slow degradation of PLA in natural environments and contamination of recycling streams due to incompatibility with other plastics highlight the need for efficient recycling techniques. This study reports a sustainable closed‐loop PLA recycling strategy by hydrolysis depolymerization under moist‐solid condition and recovery of the valuable monomer lactic acid. By employing ball milling and resonance acoustic mixing, the effects of milling speed, time, ball size, alkali type, and ageing on lactate yield are investigated, to optimize the performance and the efficiency of mechanical and moist‐solid conditions in polymer chain scission without bulk heating is demonstrated. Furthermore, a bipolar membrane electrodialysis process for conversion of lactate to lactic acid is integrated with simultaneous NaOH production, enhancing the sustainability of the recycling process. Life‐cycle assessment (LCA) is employed to assess the environmental impact of such PLA recycling approach for lactic acid production and provide recommendations for future improvements. This work demonstrates the effectiveness and environmental benefits of mechanically induced moist‐solid chemical recycling and integration with advanced membrane separation processes, offering a promising pathway for sustainable PLA waste management and lactic acid production.

## Graphical abstract

In this study, an innovative polylactic acid recycling process by integration of mechanically induced moist‐solid hydrolysis depolymerization and further recovery of lactic acid vis bipolar membrane electrolysis is reported. In‐depth life‐cycle assessment is benchmarked to conventional waste management and lactic acid production, highlighting the environmental benefits of this process.

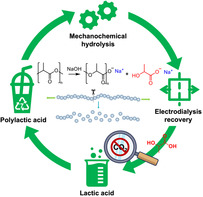

## 1 Introduction

Over the past 15 years, bioplastics have emerged as promising alternatives to traditional petroleum‐based plastics in applications such as food and beverages, 3D printing, etc.^[^ ^1^ ^]^ Derived from starch, poly(lactic acid) (PLA) is increasingly favored for its potential to mitigate environmental impact.^[^ ^2^, ^3^ ^]^ While PLA is biodegradable, it only breaks down under industrial composting conditions (e.g., high temperature and controlled humidity). In addition, when PLA is mixed with other plastics (especially polyethylene terephthalate (PET)) in recycling streams, it can contaminate the process because it has different melting points and properties.^[^ ^4^ ^]^ Therefore, going forward, it is important to separate and recycle PLA, especially when implementing in a closed‐loop process, permitting retaining the monomer feedstock in the process for higher atom efficiency.

**Figure 1:**
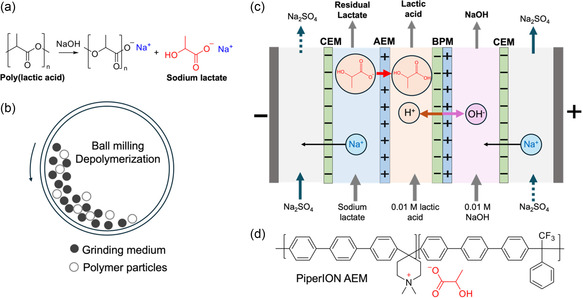
Depolymerization of PLA and recovery of lactic acid. a) Depolymerization of poly(lactic acid) via alkaline hydrolysis to sodium lactate. b) Schematic diagram of ball‐milling process. c) Bipolar membrane electrodialysis (BMED) process for conversion of sodium lactate to lactic acid and production of NaOH simultaneously. Key steps include 1) water dissociation by bipolar membrane (BPM) to acid and base; 2) transport of lactate through AEM to acid chamber; and 3) neutralization to lactic acid. CEM: cation‐exchange membrane. d) Structure of PiperION anion‐exchange membrane that selectively transport lactate anions.

An attractive strategy for PLA recycling is to depolymerize it back into the monomer lactic acid, the production of which accounts for 50% of the PLA production cost due to the complex fermentation and purification processes required.^[^ ^1^ ^]^ Recovery of lactic acid from chemical depolymerization of PLA is more favorable compared to the production from the costly fermentation route.^[^ ^5^ ^]^ Such chemical depolymerization process typically involves acid‐ or base‐catalyzed hydrolysis.^[^ ^6^, ^7^, ^8^ ^]^ Under acidic conditions, the dominating process is a chain‐end scission, whereby the terminal hydroxyl group is activated by protonation and is hydrolyzed directly to lactic acid. In contrast, base tends to initiate a random chain scission process *via* a backbiting reaction to generate lactide, which is subsequently hydrolyzed to lactate (**Figure** **1**a). Biocatalysts have also been demonstrated to effectively breakdown PLA polymer chains.^[^ ^9^, ^10^, ^11^, ^12^ ^]^ These methods, however, proceed in aqueous solution and therefore necessitate the transformation of PLA into either an emulsion or a composite to assist the depolymerization. In such cases, limitation lies in the low efficiency and lack of lactic acid (LA) recovery methods.

Mechanochemistry in this case offers an attractive solution,^[^ ^13^ ^]^ as it selectively cleaves the “weak” chemical bonds in these long chain polymers into monomers, under ambient pressure without external heat supply.^[^ ^14^, ^15^, ^16^, ^17^ ^]^ Ball milling is a convenient and powerful technique to apply force to polymers (Figure 1b). During the milling process, mechanically induced intensive friction (compression, shear, stroke, and collision) between balls or between balls and vessel walls are transmitted to the polymer chains, inducing chain scission reactions such as hydrolysis and hydrogenolysis.^[^ ^18^, ^19^ ^]^ These C—O bonds can then undergo nucleophilic attack to enable the complete breakdown of the polymer chain. The solid‐state reaction condition guarantees that the local reactant concentration is high enough to push the reaction equilibrium toward depolymerization. It also provides reduced reaction time, good stoichiometric control, and enhances product selectivity, revealing a huge potential in industrial applications.^[^ ^13^ ^]^ As a result, in recent years such mechanochemical techniques to chemically recycle plastics, such as polyethylene terephthalate,^[^ ^20^ ^]^ polypropylene,^[^ ^21^ ^]^ polystyrene,^[^ ^22^ ^]^ poly(α‐methyl styrene),^[^ ^23^ ^]^ etc., are gaining popularity. As for PLA, there are a handful of research studies investigating the effectiveness of mechanochemistry for accelerating the PLA depolymerization process.^[^ ^24^ ^]^ For example, Pérez‐Venegas et al. reported quantitative mechano‐enzymatic depolymerization of PLA to lactic acid using the *Humicola insolens* cutinase enzyme in moist‐solid reaction mixtures, highlighting the effectiveness of mechanically induced moist‐solid reaction in avoiding the use of toxic organic solvents.^[^ ^25^ ^]^ Peterson and co‐workers investigated how PLA crystallinity affects the degradation rate under ball‐mill grinding, showing negligible influence owing to the fast amorphization of semicrystalline PLA under mechanical forces,^[^ ^26^ ^]^ different from solution based PLA hydrolysis, in which crystalline PLA showed slower hydrolysis rate.^[^ ^27^, ^28^ ^]^ Lee et al. conducted mechanochemical methanolysis to convert PLA into methyl lactate with 98% product yield.^[^ ^29^ ^]^ They found that the ball milling facilitates sufficient physical contact and energy transfer between plastics and methanol, thereby eliminating the need for solvents and catalysts. In a more recent study by Makarov et al. effective PLA depolymerizing transesterification into alkyl lactate esters was conducted with resonant acoustic mixing (RAM), which achieved up to 94% product yield.^[^ ^30^ ^]^ These studies demonstrate the suitability of mechanochemistry in PLA depolymerization, but did not provide details on the overall sustainability assessment.

In this work, we report an innovative chemical process for recycling PLA *via* mechanically induced alkaline hydrolysis under moist‐solid reaction conditions. Operation parameters in a ball‐milling reactor were studied and their effects on the yield was quantified. As the product is in lactate form, we also integrated further recovery of lactic acid *via* bipolar membrane electrodialysis (BMED), a promising technology for energy‐efficient and environmental‐friendly separation and purification of organic acids. Compared to the conventional method involving adding hydrochloric acid to adjust the pH to acidic, which is material intensive, this approach is more economic and sustainable to separate lactate from the NaOH (Figure 1c) without adding additional reagents and producing an extra waste stream. The device diagram is supplied in Figure S1, Supporting information. As demonstrated by a few pilot scale studies,^[^ ^31^, ^32^ ^]^ electrodialysis (ED) is promising and suitable for large‐scale treatment of lactic acid due to several key advantages. The ED modules allow for straightforward capacity expansion by adding membrane stacks or parallel units. Compared to conventional separation methods, ED significantly reduces energy consumption and chemical usage, leading to lower operational costs. It also produces high‐purity lactic acid, which is essential for applications in the food and pharmaceutical industries. Moreover, ED can be easily integrated into modern bioprocessing and fermentation workflows, making it well suited for industrial‐scale operations. These benefits are further enhanced when ED is combined with bipolar membranes (BPMs), which enable the direct conversion of lactate salts into free lactic acid, reducing the need for external chemicals such as sulfuric acid or calcium hydroxide.

Despite its great potential in achieving efficient chemical conversion and separation, BMED still faces several scientific challenges: 1) commercial anion‐exchange membranes (AEM) used for organic acid separations are still limited by low flux rates and selectivity for the organic acid/organic acid anions over competing inorganic salt ions; 2) very limited studies exist in designing appropriate ion‐exchange membranes for organic acid separations^[^ ^33^ ^]^; 3) the conversion of lactate to lactic acid also requires membranes with high chemical stability, especially resistant to degradation by acidic and alkaline electrolyte. Here by integrating the recently developed poly(acryl piperidinium)‐based AEM membrane (known as PiperION, Figure 1d), we overcome some of the aforementioned challenges and achieved efficient conversion and recovery of lactate to lactic acid. Furthermore, in‐depth life‐cycle assessment (LCA) was performed based on the experimental results, and the climate change impact of such close‐loop PLA recycling to lactic acid is compared to the conventional lactic acid production from sugar fermentation. Looking forward, as PLA is preferentially achieved by ring‐opening polymerization of lactide (a cyclic diester of lactic acid), the formation of which from lactic acid can contribute up to 30% of the total cost for PLA production, future research will work toward PLA depolymerization into lactide directly under solid‐state condition.

## 2 Results and Discussion

### 2.1 Mechanical Depolymerization of PLA

The initial depolymerization conditions were conducted with 1.0 g PLA sample, with two equivalents of NaOH and water to depolymerize it. The mixture was then ball milled using a planetary ball mill with eight 10 mm ZrO_2_ milling balls in 50 mL jars, at 500 rpm for 10 min (5 min forward + 5 min reverse). Previous studies have shown that the vapor‐assisted ageing strategy is effective in the depolymerization of a wide range of biopolymers and of PET plastics.^[^ ^20^, ^25^, ^28^ ^]^ Therefore, an ageing step in 75 % relative humidity was performed immediately after the milling process. The temperature, 60 °C, is near the glass transition temperature for PLA, which is reported to be suitable for its hydrolysis reaction.^[^ ^19^, ^27^ ^]^ As shown in **Table** **1**, the sodium lactate yield was 80.21%, proving the effectiveness of such depolymerization approach. No other by‐product chemicals were observed, demonstrating the high selectivity of such mechanical‐induced hydrolysis reactions, a distinct advantage over conventional processes.^[^ ^13^ ^]^ Conceptually, mechanical energy is supplied in a highly localized manner and enables energy transfer between reactants without bulk heating, thereby allowing control over selectivity.^[^ ^34^ ^]^ We have thus used this reaction condition as the control to study the effects of several parameters, as well as optimize the sodium lactate yield through changing the parameters in milling speed, milling time, ageing time, milling ball size, as well as the base types.

**Table 1:** PLA depolymerization parameters and the corresponding Na/Li/K lactate yield.

Entry	Milling speed [rpm]		Milling time [min]		Ball size [mm]	Alkali type	Ageing time [day]	Lactate yield [%]
1	500		10		10	NaOH	3	80.21 ± 0.92a)
2	600		10		10	NaOH	3	65.44 ± 5.17
3	750		10		10	NaOH	3	87.84 ± 0.83
4	850		10		10	NaOH	3	92.06 ± 2.97
5	500		20		10	NaOH	3	92.06 ± 3.06
6	500		30		10	NaOH	3	94.76 ± 5.16
7	500		40		10	NaOH	3	85.10 ± 8.89
8	500		10		10	NaOH	1	78.75 ± 0.37
9	500		10		10	NaOH	2	76.93 ± 0.79
10	850		10		5	NaOH	3	79.46 ± 0.85
11	500		10		10	NaOH	0	15.58 ± 0.59
12	500		10		10	LiOHb)	0	4.93 ± 1.14
13	500		10		10	KOHb)	0	27.14 ± 0.40
14	500		20		10	KOHb)	1	81.83 ± 0.94
15	100 g acceleration, 10 minc)					KOHb)	1	91.01 ± 0.39
16	500		10		10	–	1	0.53
17	–					NaOH	1	51.47

a The Na/Li/K lactate yield is an average of at least two experiments.

b The different alkali used was kept the same 1:2 ratio for PLA:alkali molar equivalent.

c Reaction conditions using the Resodyn acoustic mixer.

**Figure 2:**
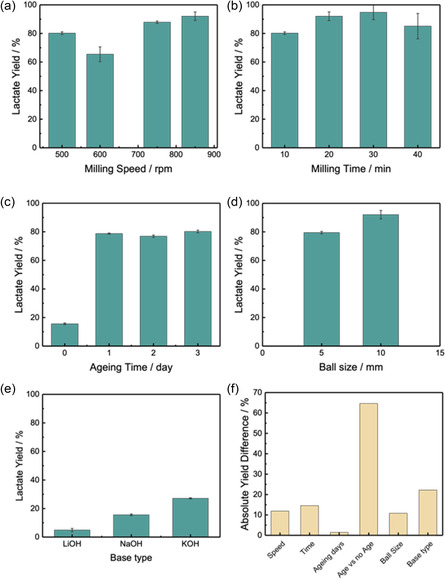
The Na/Li/K lactate production yield as a function of different reaction parameters: a) milling speed, b) milling time, c) ageing time, d) ball size, and e) base type. f) A comparison of the significance of different parameters to Na/Li/K lactate yield, represented by the absolute yield differences among each individual factors.

As shown in Table 1 and **Figure** **2**a, the lactate yield increases with increasing the milling speed, indicating that the higher kinetic energy at higher speeds is favorable for polymer chain scission. Similarly, increasing the milling time from 10 to 40 min resulted in a peak yield of 94.76% after 30 min, comparable to that reported using methanolysis and transesterification,^[^ ^29^, ^30^ ^]^ but decreasing to 85.10% after 40 min. As longer milling time also leads to a higher temperature,^[^ ^35^ ^]^ such results suggest that while prolonging the milling time may provide better exposure of reactants to mechanical forces, the thermal side effect could result in thermal degradation to undesired products, thus reducing the lactate yield. In comparison, the ageing time from 1 to 3 days showed minimal effects, with less than 5% difference in the lactate yield. However, compared to the yield with no ageing step (15.58%), it is clear that ageing has significantly increased the hydrolysis rate, in consistence with literature reports.^[^ ^20^, ^25^ ^]^ Reducing the ball size from 10 to 5 mm (matching the weight of total balls used) was found to slightly reduce the lactate yield. This distinction emphasizes the importance of mechanical forces influenced by ball size in the depolymerization process of PLA. Smaller balls, despite having a higher surface area‐to‐volume ratios that typically increase the frequency of impacts, fail to effectively deliver the required impact energy to initiate the hydrolysis reaction, thus limiting the overall depolymerization efficacy.^[^ ^24^ ^]^ In addition, presumably an interplay between mechanical and thermal effects during ball milling could also be responsible for the more efficient performance of the reaction with larger ball size.^[^ ^35^, ^36^ ^]^

Figure 2e depicts the findings from the investigation of various alkali types, as described in Table 1, entries 11–13. The experiments were performed without ageing step to show the intrinsic properties of the alkali without the hydrolysis acceleration through ageing. Despite theoretical advantages due to its lower molecular weight, which may allow for easier penetration into the PLA matrix, LiOH produced the lowest depolymerization rate at 4.93%. In contrast, KOH achieved the highest yield of 27.14%, which can be attributed to its greater basicity compared to NaOH or LiOH, which improve its ability to efficiently break down PLA's ester bonds. Since the acid dissociation constant (pK_a_) of oligomeric PLA is 3.1,^[^ ^7^ ^]^ the higher the basicity the alkali, the faster the hydrolysis kinetics are likely to be. Despite its high hydrolytic activity, NaOH produced a moderate yield of 15.58%. This finding suggests that factors such as steric hindrance or limited molecular access to PLA chains may reduce its effectiveness, preventing complete hydrolysis and limiting its potential for PLA depolymerization. Furthermore, the faster depolymerization observed with KOH compared to NaOH and LiOH highlights the importance of selecting alkalis based on their intrinsic properties to optimize depolymerization results. Two control experiments were performed: entries 16 and 17 to verify the role of alkali for catalyzing the hydrolysis process, and ball mill in initiating the hydrolysis reaction, respectively. The low lactic acid/lactate yields (0.53% and 51.47%, respectively), confirming the importance of both reaction factors.

Results from aforementioned demonstrate the importance of ageing and the choice of alkali base for hydrolysis process. The ball‐milling step serves two purposes: 1) sufficiently mixing the PLA flakes with the alkali and water, providing maximum surface contact; 2) providing mechanical force through impact to activate the polymer chain and initiate the hydrolysis process. Varying the ball‐milling parameters showed minor effect to the overall lactate yield within the range of our study (Figure 2f). Therefore, moving forward, we choose to verify the effectiveness of mechanically induced hydrolysis using another type of equipment, RAM, which is known to achieve reactivity by intensively shaking materials at a low acoustic frequency (e.g., 60 Hz), with energy input modulated through changes in the vertical acceleration of the reaction vessel (0–100 g, where g = 9.81 m s^−2^). By avoiding the need for milling media, RAM enables simplification of reaction design, avoids product contamination resulting from chipping and abrasion, as well as it eliminates the associated thermal effects, as the impact energy is lower.^[^ ^37^, ^38^ ^]^ It can also operate at larger scale than the lab‐based ball mill, as the vessels provided can process up to 500 g feedstocks. As listed in Table 1, entry 15, with 10 min mixing and 1 day ageing, the lactate yield reached 91.01 %, approaching the highest yield obtained from ball‐milling process.

### 2.2 BPM ED for Lactic Acid Recovery

A solution of 0.79 M sodium lactate (Table S1, Supporting Information), mimicking the depolymerization solution (contains sodium lactate and unreacted NaOH), was fed into the salt chambers of the BMED stack (Figure 1c), which involves several physical and chemical processes. To focus on the conversion of lactate and for process simplicity, NaOH was not included in the feed solution. The 0.01 M lactic acid and 0.01 M NaOH solutions as the initial solutions were pumped into acid and base chambers, respectively (Figure 1c and S1, Supporting Information). When the applied current density is larger than limiting current density, water dissociation happened at the interface of the BPM due to the heightened resistance of the depleted junction and a subsequently increased electric field across the junction, resulting in the generation of protons and hydroxide ions to help sustain the current flow.^[^ ^39^ ^]^ Simultaneously, lactate anions transport through AEM membrane to the acid chamber and react with produced protons to form lactic acid. Na^+^ ions transport through cation‐exchange membrane (CEM) to the base chamber and combine with OH^−^ to form NaOH. In addition to the aforementioned two main steps, there are also some secondary transport processes which may influence the lactic acid and NaOH production efficiency (Figure S1, Supporting Information). For example, hydrated ions (Na^+^ and lactate) carry water molecules as they migrate through the membrane, which can reduce acid and base concentrations. In addition, co‐ions (the same charge as the fixed functional groups within the membrane) would leak into acid or base chambers due to concentration diffusion. Co‐ions leakage could also occur in BPM. Both would influence the purity of the lactic acid and NaOH products. Ion‐exchange membranes with higher perm‐selectivity can be developed to minimize the water migration and co‐ion leakage. Overall, water dissociation reactions and lactate anion transport are the two most important steps in BMED process for lactic acid recovery.

**Figure 3:**
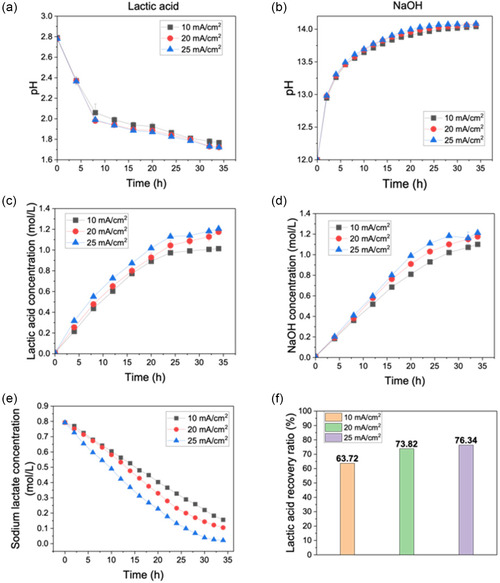
Performance of BMED in lactic acid and NaOH recovery: pH change in a) lactic acid chamber and b) NaOH chamber; concentration change in c) lactic acid chamber and d) NaOH chamber; the remaining unrecovered e) sodium lactate concentration and f) lactic acid recovery ratio.

The pH change in the acid and base chambers as a function of time is shown in **Figure** **3**a,b. After 34 h of testing, the pH in the acid chamber decreased from ≈2.8 to 1.7, while the pH in the base chamber rose above 14. The final pH of the acid solution corresponds to lactic acid concentrations of 1.01 m at 10 mA cm^−^^2^, 1.17 m at 20 mA cm^−^^2^, and 1.21 m at 25 mA cm^−^^2^ (Figure 3c), while that of base solution corresponds to NaOH concentrations of 1.1 m at 10 mA cm^−^^2^, 1.17 m at 20 mA cm^−^^2^, and 1.21 m at 25 mA cm^−^^2^ (Figure 3d). This phenomenon indicates that increasing the current density could only slightly increase the lactic acid and base production. Higher current density could lead to faster water dissociation rate and ion transport through membranes. But under higher current, water supply becomes insufficient, leading to incomplete water dissociation and a lower‐than‐expected acid–base production rate. It can also be seen from Figure 3c,d that the acid and base production became slow with longer operating times. This can primarily result from the back diffusion of H^+^ and OH^−^ ions through the membrane, driven by the increasing pH gradient.

The variation of sodium lactate concentration in feed depolymerized lactate solution under different current densities was also examined (Figure 3e). The concentration of sodium lactate dropped to a very low level after 34 h. Especially under 25 mA cm^−2^, the concentration was only 0.022 M (Table S1, Supporting Information). This means that a large part of sodium lactate was recovered as lactic acid. The recovery rate shown in Figure 3f indicates that the recovery ratio can reach 76.34% at a current density of 25 mA cm^−^^2^. In addition to some remaining in the original solution, a small portion of lactate ions was discovered to leak into the electrode chamber due to concentration difference (Figure S2, Supporting Information).

**Figure 4:**
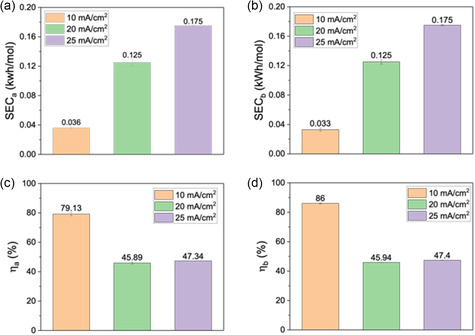
Specific energy consumption (SEC) and current efficiency (η) of the BMED: lactic acid: a) SEC_a_, b) SEC_b_, c) η_a_; NaOH: d) η_b_.

Finally, to further explore the optimal current density for lactic acid recovery efficiency, the specific energy consumption (SEC) for acid/base production and current efficiency were calculated. As shown in **Figure** **4**a,b, the current density at 10 mA cm^−2^ could achieve the lowest SEC (0.036 kWh mol^−1^ LA and 0.033 kWh mol^−1^ NaOH), which was nearly one fifth of the SEC at 25 mA cm^−2^. When the current density was 10 mA cm^−2^, the current efficiency was around 80% (nearly twice the values of the other two current densities), meaning that the fraction of the total electrical charge that contributes to the ion transport was much higher. Therefore, an applied current density at 10 mA cm^−2^ was the best one for lactic acid recovery. Although there are not many previous studies on lactic acid recovery *via* ED, a benchmark of our system to the literature shows that our performance is among the highest in terms of high acid and base recovery, as well as low energy consumption (Table S2, Supporting Information).^[^ ^34^, ^35^, ^36^ ^]^ In future studies, the energy consumption per unit of acid/base production can be further decreased through increasing the membrane triplets between the ED stack due to more effective voltage distribution, lower resistance, and improved electrode utilization.

### 2.3 Life‐Cycle Impact Assessment

LCA (ISO14040‐44)^[^ ^40^ ^]^ has been applied to predict the life‐cycle global warming potential (GWP)^[^ ^41^ ^]^ and other environmental impacts^[^ ^42^ ^]^ of the recycling of waste PLA systems, in order to compare its environmental sustainability against existing systems.^[^ ^43^, ^44^ ^]^ We used the lab experimental data for two base scenarios: Table 1, entry 6 as the optimized ball‐mill system (Scenario 1), and Table 1, entry 15 as the unoptimized RAM system (Scenario 2). The conventional lactic acid production via sugar fermentation (see Table S3–S6, Supporting Information) and business‐as‐usual PLA end‐of‐life waste management *via* incineration with energy recovery and landfill (see Table S7–S8 and Figure S3, Supporting Information) are used as benchmark systems to show the benefits of PLA recycling into the lactic acid production system.^[^ ^45^ ^]^

The new system of PLA depolymerization into lactic acid production can be evaluated for the life‐cycle impact assessment (LCIA) for the two systems: 1) to recycle 1 kg PLA and 2) to produce 1 kg lactic acid. These two systems’ LCIA will answer the following two research questions. 1) How does the LCIA of waste PLA recycling compare against business‐as‐usual plastic recycling systems (incineration with energy recovery and landfill)? 2) How does the LCIA of lactic acid production through PLA recycling compare against the conventional lactic acid production systems? The cradle‐to‐gate inventory flow of PLA recycling into the lactic acid production system has been constructed as shown in Table S9, Supporting Information, and the selected database is in Table S3, Supporting Information, the same as that of the conventional lactic acid production system. Furthermore, the embedded life‐cycle impact of 1 kg PLA has been added to consider the impact of feedstock and that of 1.152 kg lactic acid production (due to the addition of proton and hydroxyl groups onto building block during hydrolysis reaction, calculation see Table S9, Supporting Information, Scenario 1) has been subtracted because this lactic acid would displace the conventional lactic acid production to calculate the net savings in Scenario 1: optimal ball‐mill system.

**Figure 5:**
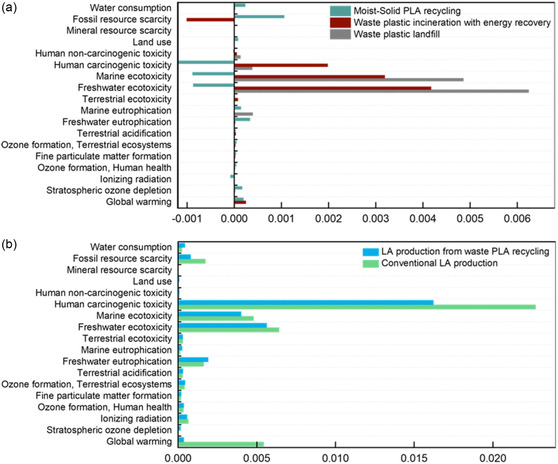
a) Normalized LCIA comparisons between PLA recycling (Scenario 1: optimized ball‐mill system), waste plastic incineration with energy recovery avoiding equivalent natural gas and waste plastic landfill systems. b) Normalized LCIA comparisons between lactic acid (LA) production through PLA recycling (Scenario 1: optimized ball‐mill system) and the conventional LA production systems.

**Figure** **5**a shows a comparison of the normalized results of Scenario 1: optimized ball‐mill system against the business‐as‐usual waste management systems in Figure S3, Supporting Information. It is important to note that the credits from the heat and electricity recovered through incineration has been claimed in the benchmark system. The new PLA recycling system (see Table S10, Supporting Information, for the ReCiPe Midpoint (H) LCIA) is the best option among the three choices in most categories, except water consumption, land use, freshwater eutrophication, and stratospheric ozone depletion. Its higher impacts in the categories are due to the bio‐based PLA production system. Most importantly the new PLA recycling system (Scenario 1: optimized ball‐mill system) is the most environmentally friendly option in terms of GWP and resource scarcity, shown by their negative values or environmental savings or avoided environmental impacts.

Furthermore, to compare the LCIA of lactic acid production through PLA recycling against the conventional lactic acid production systems, the inventory values are transformed to correspond to 1 kg lactic acid production. For example, Scenario 1: in optimized ball‐mill system, 1 kg lactic acid is produced from 0.86 kg PLA using 0.5 kWh electricity and 0.034 kg NaOH; its LCIA (Table S11, Supporting Information) is compared against the LCIA of the conventional lactic acid production system *via* sugar fermentation (Table S4, Supporting Information). Figure 5b shows their normalized LCIA comparisons. It can be seen that the results in both figures are comparable. Except for water consumption, land use, freshwater eutrophication, and ozone depletion, lactic acid production through PLA recycling (Scenario 1: optimized ball‐mill system) is better than the conventional lactic acid production system. Thus, from both PLA recycling and lactic acid production perspectives, the new recycling process is more environmentally benign.

**Figure 6:**
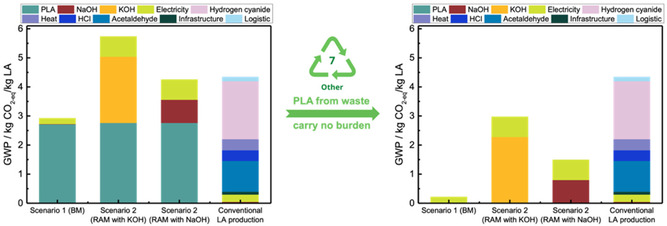
GWP dominance analysis comparing the lactic acid (LA) production systems: Scenario 1: optimized ball‐mill system; Scenario 2: unoptimized RAM system and the conventional system.

**Figure** **6** shows the GWP contribution analysis comparing the lactic acid production systems from Scenario 1: optimized ball‐mill system and Scenario 2: unoptimized RAM system and the conventional system. The raw material usage has a higher dominance than energy use or any other inventories. Sourcing PLA feedstock is the highest contributor to lactic acid production in the PLA recycling system. As more and more PLA is recycled from the waste stream and credits are accounted for, the LCIA results of PLA recycling or lactic acid production from waste PLA depolymerization would look more and more attractive compared to business‐as‐usual or conventional systems. For example, if PLA is sourced from waste materials carrying no burden, a 92% reduction in GWP in Scenario 1: optimized ball‐mill system, a 65% reduction in GWP in Scenario 2: unoptimized RAM system (with NaOH) and a 48% reduction in GWP in Scenario 2: unoptimized RAM system (with KOH) is possible (Figure 6).

Clearly, KOH has a larger impact than NaOH, revealing that future upscaling must be developed with NaOH and maximize the NaOH in‐process circulation, thus reducing its fresh supply for the process. Electricity has a negligible impact. However, a reduction in electricity consumption is possible. Scenario 1: optimized ball‐mill system has a lower carbon footprint than the conventional system for lactic acid production. Scenario 2: unoptimized RAM system with KOH as a reagent has a higher GWP than the conventional system for lactic acid production, while the unoptimized RAM system with NaOH as a reagent has a slightly lower GWP than the conventional system for lactic acid production. To note here that the RAM process was not optimized, and the GWP values could be reduced during further optimization. Lactic acid produced from PLA sourced from waste materials carrying no burden to the environment can reduce the GWP of conventional lactic acid production across all scales.

Furthermore, a future system with renewable electricity^[^ ^46^, ^47^ ^]^ uses is evaluated to show the GWP reduction in PLA recycling/lactic acid from PLA production impacts in Scenario 1: optimized ball‐mill system considering PLA environmental footprints. And, 10 000 Monte Carlo simulation runs are performed for this system varying the amount of sodium hydroxide by a ±10% with a normal distribution.^[^ ^48^ ^]^ The results of this uncertainty analysis on the normalized ReCiPe (M) (H) significant categories are shown in Figure S4, Supporting Information. To compare between the life‐cycle impact categories, their normalized (dimensionless values) instead of characterized impacts are shown. Apart from fossil resource scarcity and freshwater eutrophication, other life‐cycle impact categories including GWP are found to be fairly certain or robust. With renewable electricity uses and avoided impacts from lactic acid production, a net GWP saving with a mean value of −1.8 kg CO_2_e/kg PLA, is obtained, making the net GWP saving plausible for the new system. This study for the first time analyzed the LCIA to demonstrate comprehensively the environmental incentives from both PLA recycling and lactic acid production perspectives using the mechanically induced moist‐solid remanufacturing technologies.

## 3 Conclusion

In this work, we report an innovative two‐step chemical process for recycling of PLA by integration of mechanically induced moist‐solid depolymerization hydrolysis and further recovery of lactic acid via BMED. Ball‐milling process coupled with vapor‐assisted ageing was highly effective for the hydrolysis of PLA into lactate. Results have shown varying the milling conditions has negligible effects on the lactate yield, while having the ageing step has proven to be crucial. The optimized parameters are repeated at larger scale (>10 g PLA capacity) in a RAM reactor and achieved 91.01% ± 0.39% lactate yield, demonstrating the potential for scaling up applications. While this protocol uses a strong base such as NaOH, the downstream ED process can effectively recover the lactic acid form sodium lactate and generate NaOH simultaneously, diminishing its environmental impact. Finally, our LCA study demonstrates the environmental friendliness of such process both as a recycling method and as a lactic acid production approach. The GWP value for the optimized ball‐mill system showed a 33% reduction over conventional lactic acid production system. And if PLA is sourced from waste materials carrying no burden to the environment in the future, a significant 92% reduction can be achieved.

## Conflict of Interest

The authors declare no conflict of interest.

## Supporting information

Supplementary Material

## Data Availability

The data that support the findings of this study are available from the corresponding author upon reasonable request.
