# Supplementary material for: Process Integration and Life‐Cycle Assessment of Moist‐Solid Hydrolysis of Polylactic Acid with Lactic Acid Recovery via Electrodialysis
Source: ChemSusChem. 2025 May 20;18(14):e202500503. doi: 10.1002/cssc.202500503 (PMC12270370; doi:10.1002/cssc.202500503)
Supplement: Supplementary file 1 — Supplementary Material [file CSSC-18-e202500503-s001.pdf]

Supporting Information of

Process Integration and Life-cycle Assessment of Moist-Solid Hydrolysis of Polylactic Acid with Lactic Acid Recoveryvia Electrodialysis

Hui Luo,*[a] Dingchang Yang,[b] Jhuma Sadhukhan,[a] VerdeluzCostica,[a] Robert Dorey,[a] Qilei Song,[b] Maria-Magdalena Titirici[b]

Experimental Details

PLA depolymerisation

All chemicals were purchased from commercial sources (Merck) and were used as received. Post consumer wastePLA cups (from Vegware) were washed with detergent and DI water, dried, and cut into small 2 mm * 3 mm flakes and used as the feedstock for depolymerisation. The milling process was carried out with a Fritsch Planetary Micro Mill Pulverisette 7. A mixture of 1 g of PLA flakes, LiOH, NaOH and NaOH (2 equiv.) and 0.5 g DI water was ball milled in a 50 mL ZrO_2jar using eight 10 mm ZrO_2 balls for the respective time under conditions in **Table 1**.After ball mill, the contents in the jar were transferred into small vials, which then were placed in aging chambers with the relative humidity levels set to 75 % (sat. NaCl). After ageing at 60 °C for the respective reaction time, each reaction mixture was suspended in 27.8 mL of DI water.

For the RAM process, a ResodynLabRAM II instrument was used. 13.927 g PLA flakes was mixed with 2 equiv. $KOH{\cdot}H_2O$ and 7 mL DI water. The instrument was set to run at 100 g acceleration for 10 min. After reaction, the mixture was transferred to a 50 mL plastic jar (made from polypropylene) and placed in an oven at 60 °C for 24 hours. After ageing, the reaction mixture was diluted with DI water to an estimated concentration of 1 M for sodium lactate, ready for electrodialysis.

The Agilent 1260 Infinity II LC high-performanceliquid chromatography (HPLC)system was used for lactic acid quantification. This system used an Aminex HPX-87H column at40°C and a refractive index detector at 35°C. The mobile phase consisted of 5 mM H_2SO_4flowing at 0.4 mL min^{-1} and an injection volume of 1 μL. Samples for HPLC were prepared bymixing 200 μL of reaction products with 200 μL of 0.55M H_2SO_4, following filtration through asyringe filter to remove particulate matter

Bipolar membrane electrodialysis

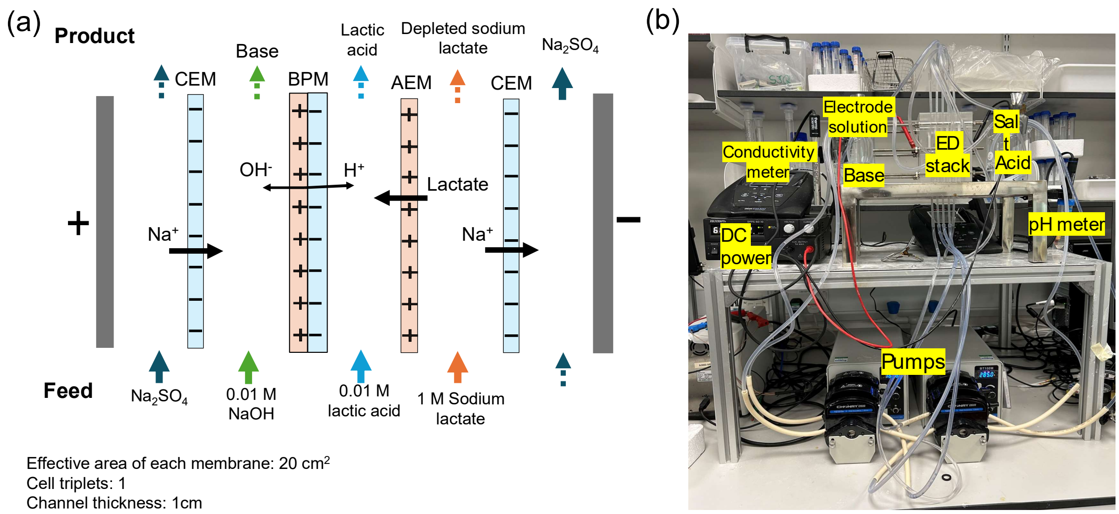

Figure S1 Experimental setup of the BMED for LA and NaOH recovery.(a) Schematic diagram of BMED process. (b) Picture of BMED setup.

Table S1. Solution chemical composition comparison of the initial simulated ED solution and the recovered streams.

Chamber	Chemical	Initial Concentration (mol/L)	Result concentration (mol/L)	Volume (mL)	Flow rate (mL/min)	Current density (mA/cm^2)
**Salt**	Sodium lactate	0.79	0.156 (10 mA/cm^2)<br>0.105 (20 mA/cm^2)<br>0.022 (25 mA/cm^2)	400	80	10 or 20 or 25
**Acid**	Lactic acid	0.01	1.01 (10 mA/cm^2)<br>1.17 (20 mA/cm^2)<br>1.21 (25 mA/cm^2)	200	80	
**Base**	NaOH	0.01	1.1 (10 mA/cm^2)<br>1.17 (20 mA/cm^2)<br>1.21 (25 mA/cm^2)	200	80	

**Electrode**	Na_2SO_4	0.3	0.3	400	80	

In this study, a laboratory-scale bipolar membrane electrodialysis (BMED) stack was utilized, consisting of a single triplet of membranes arranged in parallel: an anion exchange membrane (AEM - PiperION® Anion Exchange Membrane), a cation exchange membrane (CEM - Nafion®), and a bipolar membrane (BPM - Fumasep FBM). Each membrane had an effective area of 20 cm^2, separated by a 1 cm-thick channel between adjacent membranes (**Figure S1a**). The BMED stack was operated in galvanostatic mode, powered by a DC supply from ShenzhenshiZhicheng Electronics Tech. Co., LTD. Voltage readings were recorded every 1 hour, along with pH measurements of the acid and base solutions at the same intervals. In the BMED system, the depolymerized solution and 0.3 M Na_2SO_4 solution were circulated through the salt and electrode chambers, while 0.01 M lactic acid and 0.01 M NaOH was circulated through the acid and base chambers, respectively. This circulation was maintained using peristaltic pumps from Baoding Chuangrui Precision Pump Co. A photograph of the BMED setup for lactic acid and NaOH production is shown in **Figure S1b**.The volumes of feed solutions to salt and electrode chambers were 400 mL, solutions pumped into acid and base chambers were 200 mL. All experiments were conducted for a fixed duration of 34 hours. Samples were collected every two hours to assess the concentrations of lactic acid and NaOH. In the final products, the concentration of lactic acid was measured by using high-performance liquid chromatography (HPLC)and the concentration of NaOH was measured by titration method.

Specific Energy Consumption and Current Efficiency

The specific energy consumption (SEC_i [kWh kg^{-1}]), defined as the electrical work required to produce one mole of species *i*, was calculated using Eq. 1:

$$\mathrm{SEC_i} = \frac{IA\int_0^{\tau} U(\tau')d\tau'}{C_i(\tau)V_i(\tau) - C_i(0)V_i(0)} \#(1)$$

Where $U(\tau')$ [V] represents the applied voltage across the BMED stack at time $\tau'.I$ [A m^{-2}] denotes the current density.$\Delta\tau$is the time interval [s] for voltage recording. The voltage integral was numerically approximated using a first-order Backward-Euler scheme. For the specific energy consumption (SEC) normalized by acid production, $C_i(\tau)$ [mol L^{-1}] and $C_i(0)$ [mol L^{-1}] represent the acid concentrations at times time τ and 0, respectively, while

$V_i\,(\tau)$ [L] and $V_i\,(0)$ [L] correspond to the acid volumes at those same times. Similarly, for SEC normalized by base production, $C_i\,(\tau)$ [mol L^{-1}] and $C_i\,(0)$ [mol L^{-1}] denote the base concentrations at time τ and 0, with $V_i\,(\tau)$ [L] and $V_i\,(0)$ [L] representing the respective base volumes.

Finally, the overall current efficiency (η [-]) of the BMED system, which quantifies the effectiveness of current utilization across the membrane and electrolyte assembly, can be determined using Eq. 2:

$$\eta = \frac{F\left[C_i\left(\tau_f\right)V_i\left(\tau_f\right) - C_i(0)V_i\,(0)\right]}{IA\,\tau_f n} \#(2)$$

where n [-] is the number of the cell triplets in the BMED stack, $C_i\left(\tau_f\right)$ [mol L^{-1}] is the concentration of the product acid or base at the end of each test (i.e., at time τ_f).

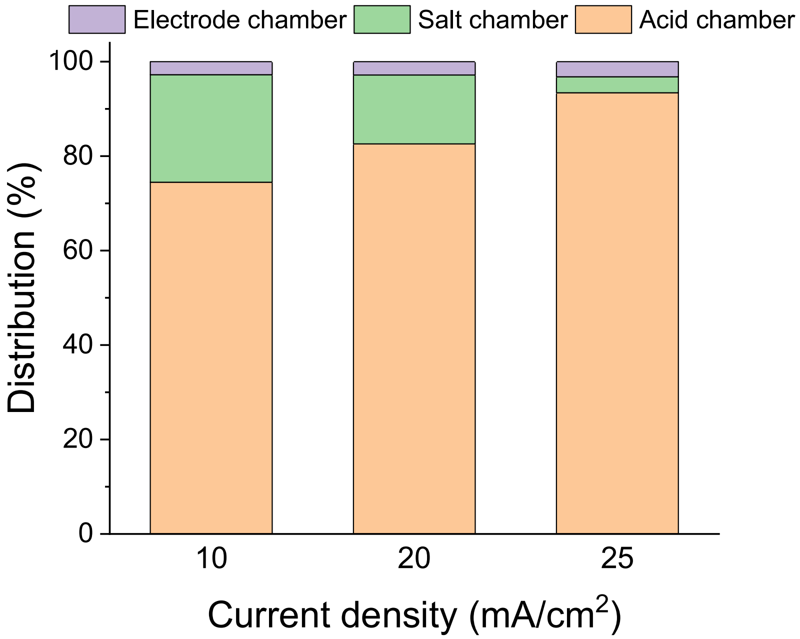

Figure S2 Distribution of lactate ions or lactic acid in acid, salt and electrode chambers under different current densities at the end of experiment.

Feed solution	Initial concentration of lactate in salt chamber (M)	Initial concentrations of solutions in acid/base chamber (M)	Volume ratio of feed to product	By products	Current efficiency (%)	Energy consumption (for BPED alone)	Ref
Sodium lactate	1 M lactate	0.0087M HLa/0.01M NaOH	2/1	HLa 1.0-1.2 M NaOH 1.1-1.2 M (10-25 mA/cm2)	Acid: 47-80 Base: 47-86	0.036-0.175kWh/mol HLa 0.033-0.175 kWh/mol NaOH	This work
Simulated beet molasses (2 wt% of lactic acid and 2 wt% of citric acid)	0.22 M HLa	0.009 M HLa/0.01 M NaOH	1:1/5:1/10:1	HLa 0.23–1.7 M NaOH 0.27–1.16 M (6–12 V/cm)	Acid: 30-80	0.113-0.0.2 kWh/mol HLa	[1]
Fermentation broth	0.075 M lactate	0.1 M HLa/0.01 M NaOH	NA	HLa 0.8-1.4 M (10-40 mA/cm^2)	NA	NA	[2]
Fermentation broth	0.27 M lactate	0.2 M HLa/0.1 M NaOH	5/1	HLa 0.67-1 M NaOH 1.4–1.75 M (20-50 mA/cm^2)	NA	NA	[3]

Table S2. Comparison of the data on lactic acid and base recovery by BMED.[1–3]

Life Cycle Assessment

SimaPro 9.4 is applied to generate all the LCA study results.LCA comprises four interactive stages, goal and scope definition, inventory analysis, impact assessment and interpretation. The functional unit, system boundaries, and systems are defined in the goal and scope definition.Here, our primary focus is the GWP evaluation. At the same time, we will also present the environmental profiles of selected cases of a range of categories using the globally accepted/respected standard life cycle impact assessment (LCIA) method, ReCiPe

Midpoint (Hierarchist, H).[4–6] This study shows the new PLA mechanochemical depolymerisation into lactic acid (LA) system evaluations using the IPCC GWP characterisation factors[7] as well as for various other categories.[8,9] The new system's LCA must consider the future renewable energy provision for feasibility, similar to.[9] The functional unit is a 1 kg PLA recycling and 1 kg LA production. The choice of these two functional units answers two research questions: 1) which is the environmentally most benign or efficient PLA recycling method or how the LCIA of mechanochemical PLA remanufacturing into LA systems compares against business-as-usual (BAU) systems, incineration with energy recovery and landfill and 2) how the LCIA of LA production from mechanochemical PLA remanufacturing compares against conventional LA production systems.

The foreground system's inventory flows come from the experimentally developed mechanochemical PLA remanufacturing into LA systems. The background systems comprise cradle-to-grave systems to support all the material and energy uses of the foreground system. The same system boundary principles are applied to the benchmark systems. The up-to-the-gate point has been considered for the systems. The scope of the systems under consideration is global meaning that the global supply chains are considered, while the plat location is assumed to be in Europe. In addition, cut-off systems are drawn to show improvements in the environmental profiles of the new system. Hot spots are analysed for the various systems under consideration. As mentioned above, GWP is the main life cycle impact considered, while ReCiPe Midpoint (H) is applied as appropriate to show any potential tradeoffs. Life cycle inventory data are extracted from Ecoinvent 3.10. The background life cycle inventory datasets range Europe (RER), the rest of the world (RoW) or global (GLO) in the cut-off (i.e., recycle streams carry no impact) systems, as shown in **Table S2**. In case of the unavailability of RER data, RoW data have been used. In case of the unavailability of both RER and RoW data, GLO data are applied.

This section shows the life cycle impact assessment (LCIA) of the conventional lactic acid production benchmark systems considered in this study. The selected Ecoinvent 3.10 databases are included in **Table S3**. The lactic acid production system's ReCiPe Midpoint (H) LCIA is shown in **Table S4**. Its life cycle inventory data are shown in **Table S5**, the variable deviations are used for uncertainty analysis in **Table S6**. The uncertainty analyses in **Table S6** show that the highest variations occur with water consumption, while Global warming potential (GWP) has one of the least standard deviations among all categories. The hotspots

are the sourcing of the two reactants, acetaldehyde and hydrogen cyanide, replacing which by alternative remanufacturing system seems to be a sustainable way forward.

Table S3. Selected Ecoinvent 3.10 databases.

Inventory	Ecoinvent database name
Electricity	Electricity, medium voltage {RER}| market group for | Cut-off
Sodium hydroxide	Sodium hydroxide, without water, in 50% solution state {GLO}| market for | Cut-off
Potassium hydroxide	Potassium hydroxide {GLO}| market for | Cut-off
Waste plastics incineration	Waste plastic, mixture {RoW}| treatment of waste plastic, mixture, municipal incineration | Cut-off
Waste plastics landfill	Waste plastic, mixture {RoW}| treatment of waste plastic, mixture, sanitary landfill | Cut-off
PLA	Polylactide, granulate {GLO}| market for | Cut-off
LA	Lactic acid {RER}| production | Cut-off

The PLA inventory data corresponds to the aggregated data[10] from the world's largest NatureWorks PLA plant. The data includes maize production, energy use, transport, infrastructure, and wastewater treatment. The incorporation of the PLA life cycle inventory dataset in its remanufacturing into the LA production system considers its impact on the LA's environmental footprint. This system of incorporating the PLA life cycle inventory dataset in its recycling into the LA production system has been shown to represent Ecoinvent's APOS (at the point of substitution: recycling streams carry environmental footprints) and consequential (future) systems. The process "lactic acid, at plant, RER" is modelled for the production of lactic acid from acetaldehyde in Europe.[10] In the Ecoinvent database, the raw materials are modelled with a stoichiometric calculation, emissions are estimated, and energy use, infrastructure and transport are calculated with their standard values.[10] The LA life cycle dataset is the benchmark to compare the PLA recycling into the LA production system.

Table S4. ReCiPe Midpoint (H) LCIA of 1 kg Lactic acid {RER}| production | Cut-off (1,4-DCB is the 1,4-dichlorobenzene equivalent representing the toxicity categories). The overall impact column has been highlighted, while the other columns represent the dominance analysis.

Impact category	Unit	Total	Lactic acid {RER}| production | Cut-off, U	Acetaldehyde {GLO}| market for | Cut-off, U	Chemical factory, organics {RER}| construction | Cut-off, U	Hydrochloric acid, without water, in 30% solution state {RER}| market for | Cut-off, U	Hydrogen cyanide {RER}| market for hydrogen cyanide | Cut-off, U	Sodium hydroxide, without water, in 50% solution state {GLO}| market for | Cut-off, U	water, decarbonised {CH}| market for water, decarbonised | Cut-off, U	water, decarbonised {DE}| market for water, decarbonised | Cut-off, U	water, decarbonised {ES}| market for water, decarbonised | Cut-off, U	water, decarbonised {FR}| market for water, decarbonised | Cut-off, U	water, decarbonised {GB}| market for water, decarbonised | Cut-off, U	Electricity, medium voltage {RER}| market group for | Cut-off, U	Heat, from steam, in chemical industry {RER}| market for heat, from steam, in chemical industry | Cut-off, U	Inert waste, for final disposal {CH}| market for inert waste, for final disposal | Cut-off, U
Global warming	kg CO_2 eq	4.35E+00	1.51E-01	1.06E+00	9.81E-02	3.66E-01	2.00E+00	4.44E-02	3.73E-09	1.91E-05	2.77E-06	4.37E-06	4.63E-07	2.51E-01	3.82E-01	4.50E-03
Stratospheric ozone depletion	kg CFC11 eq	9.53E-07	0.00E+00	1.87E-07	4.79E-08	4.02E-07	5.55E-08	4.72E-08	3.00E-15	9.76E-12	1.27E-12	3.01E-12	2.35E-13	1.18E-07	9.28E-08	2.93E-09
Ionizing radiation	kBq Co-60 eq	3.11E-01	0.00E+00	3.56E-02	1.53E-02	1.07E-01	1.14E-02	4.85E-03	3.23E-09	2.78E-06	1.13E-06	9.66E-06	2.10E-07	1.28E-01	9.07E-03	1.69E-04
Ozone formation, Human health	kg NOx eq	6.92E-03	2.16E-04	2.45E-03	3.21E-04	8.31E-04	2.03E-03	1.16E-04	1.35E-11	3.48E-08	1.02E-08	1.66E-08	1.26E-09	4.52E-04	4.61E-04	3.55E-05
Fine particulate matter formation	kg PM2.5 eq	4.28E-03	0.00E+00	1.39E-03	2.37E-04	8.12E-04	1.07E-03	9.56E-05	5.84E-12	1.46E-08	6.01E-09	6.94E-09	4.95E-10	3.72E-04	2.92E-04	9.21E-06
Ozone formation, Terrestrial ecosystems	kg NOx eq	7.31E-03	3.48E-04	2.55E-03	3.28E-04	8.45E-04	2.15E-03	1.17E-04	1.38E-11	3.55E-08	1.04E-08	1.70E-08	1.28E-09	4.56E-04	4.73E-04	3.62E-05
Terrestrial acidification	kg SO_2 eq	1.17E-02	0.00E+00	3.23E-03	4.96E-04	2.14E-03	3.88E-03	1.61E-04	1.29E-11	3.66E-08	1.47E-08	1.55E-08	1.21E-09	9.25E-04	8.64E-04	2.03E-05
Freshwater eutrophication	kg P eq	1.06E-03	0.00E+00	2.41E-04	1.15E-04	2.69E-04	1.27E-04	2.17E-05	2.43E-11	5.84E-08	8.04E-09	2.44E-08	1.38E-09	2.47E-04	3.70E-05	3.05E-07
Marine eutrophication	kg N eq	6.11E-04	0.00E+00	2.01E-05	5.51E-06	2.62E-05	5.37E-04	2.22E-06	2.72E-11	4.41E-08	8.67E-09	2.85E-08	1.57E-09	1.75E-05	2.52E-06	3.17E-08
Terrestrial ecotoxicity	kg 1,4-DCB	4.42E+00	1.16E-03	9.73E-01	1.02E+00	1.28E+00	2.90E-01	8.46E-02	1.67E-08	2.71E-05	6.24E-06	1.95E-05	1.04E-06	1.79E-01	5.47E-01	4.45E-02
Freshwater ecotoxicity	kg 1,4-DCB	1.62E-01	3.75E-04	3.17E-02	6.18E-02	3.95E-02	1.35E-02	2.46E-03	3.24E-10	1.16E-06	1.43E-07	3.87E-07	2.30E-08	1.04E-02	1.77E-03	6.74E-05
Marine ecotoxicity	kg 1,4-DCB	2.09E-01	3.01E-05	4.14E-02	7.81E-02	5.10E-02	1.79E-02	3.20E-03	4.43E-10	1.57E-06	1.93E-07	5.20E-07	3.15E-08	1.39E-02	3.05E-03	1.15E-04
Human carcinogenic toxicity	kg 1,4-DCB	2.34E-01	5.05E-04	4.84E-02	6.27E-02	4.92E-02	4.49E-02	3.65E-03	5.17E-09	9.28E-06	1.72E-06	5.39E-06	3.03E-07	1.81E-02	6.06E-03	3.59E-04
Human non-carcinogenic toxicity	kg 1,4-DCB	2.92E+00	8.70E-03	6.86E-01	8.31E-01	7.26E-01	2.38E-01	5.17E-02	4.49E-08	9.12E-05	1.52E-05	4.69E-05	2.64E-06	3.15E-01	6.55E-02	2.03E-03
Land use	m^2a crop eq	5.47E-02	0.00E+00	1.56E-02	1.17E-02	1.35E-02	2.94E-03	1.02E-03	6.92E-10	1.04E-06	2.08E-07	6.00E-07	5.39E-08	5.97E-03	3.30E-03	6.06E-04
Mineral resource scarcity	kg Cu eq	1.05E-02	0.00E+00	1.87E-03	4.67E-03	2.54E-03	8.60E-04	1.41E-04	2.58E-11	4.50E-08	9.42E-09	3.43E-08	1.63E-09	2.79E-04	8.92E-05	8.10E-06
Fossil resource scarcity	kg oil eq	1.70E+00	0.00E+00	6.44E-01	2.38E-02	1.17E-01	7.05E-01	1.10E-02	1.37E-09	5.39E-06	8.73E-07	1.63E-06	1.73E-07	6.73E-02	1.28E-01	2.55E-03
Water consumption	m3	6.27E-02	1.68E-02	1.18E-02	1.34E-03	1.34E-02	1.30E-02	1.16E-03	1.35E-07	2.12E-04	4.27E-05	1.41E-04	7.76E-06	4.23E-03	3.99E-04	1.03E-04

Table S5. Inventory data for Lactic acid {RER}| production | Cut-off, U in Ecoinvent 3.10.

Lactic acid {RER}| production | Cut-off, U	1	kg	Distribution	2SD
**Input (material)**				
Water, cooling, unspecified natural origin, RER	0.04393	m^3	Lognormal	1.2152
Acetaldehyde {GLO}| market for | Cut-off, U	0.54171	kg	Lognormal	1.2152
Chemical factory, organics {RER}| construction | Cut-off, U	7.32E-10	p	Lognormal	2.1157
Hydrochloric acid, without water, in 30% solution state {RER}| market for | Cut-off, U	0.60819	kg	Lognormal	1.2152
Hydrogen cyanide {RER}| market for hydrogen cyanide | Cut-off, U	0.33241	kg	Lognormal	1.2152
Sodium hydroxide, without water, in 50% solution state {GLO}| market for | Cut-off, U	0.033788	kg	Lognormal	1.2152
water, decarbonised {CH}| market for water, decarbonised | Cut-off, U	0.000134	kg	Lognormal	1.2152
water, decarbonised {DE}| market for water, decarbonised | Cut-off, U	0.210337	kg	Lognormal	1.2152
water, decarbonised {ES}| market for water, decarbonised | Cut-off, U	0.042403	kg	Lognormal	1.2152
water, decarbonised {FR}| market for water, decarbonised | Cut-off, U	0.139429	kg	Lognormal	1.2152
water, decarbonised {GB}| market for water, decarbonised | Cut-off, U	0.007697	kg	Lognormal	1.2152
**Input (energy)**				
Electricity, medium voltage {RER}| market group for | Cut-off, U	0.60953	kWh	Lognormal	1.0502
Heat, from steam, in chemical industry {RER}| market for heat, from steam, in chemical industry | Cut-off, U	3.6608	MJ	Lognormal	1.0502
**Output (air)**				
Acetaldehyde	0.001083	kg	Lognormal	1.0502
Carbon dioxide, fossil	0.15108	kg	Lognormal	1.0502
Water/m3	0.017178	m^3	Lognormal	1.4918
**Output (water)**				
Acetaldehyde	0.0026	kg	Lognormal	2.1157
BOD5 (Biological Oxygen Demand)	0.004533	kg	Lognormal	1.4918
Chloride	0.029565	kg	Lognormal	2.1157
COD (Chemical Oxygen Demand)	0.004533	kg	Lognormal	1.4918
DOC, Dissolved Organic Carbon	0.001418	kg	Lognormal	1.4918
Sodium	0.019428	kg	Lognormal	4.0332
TOC, Total Organic Carbon	0.001418	kg	Lognormal	1.4918
Water, RER	0.027152	kg	Lognormal	1.4918
**Outputs (waste to technosphere)**				
Inert waste, for final disposal {CH}| market for inert waste, for final disposal | Cut-off, U	0.58889	kg	Lognormal	1.2152

Table S6. Statistical analyses of ReCiPe Midpoint (H) categories of Lactic acid {RER}| production | Cut-off, U due to deviations of the variables in Ecoinvent 3.10.

Impact category	Unit	Mean	Median	SD	CV	2.50%	97.50%	SEM
Water consumption	m^3	0.061764	0.073053	0.488474	790.8701	-0.950112	0.97694406	0.00488474
Terrestrial ecotoxicity	kg 1,4-DCB	4.418813	4.184653	1.173391	26.55444	2.9022228	7.4398889	0.01173391
Terrestrial acidification	kg SO_2 eq	0.011736	0.011675	0.000847	7.219089	0.0102871	0.013566469	8.47E-06
Stratospheric ozone depletion	kg CFC11 eq	9.54E-07	9.43E-07	1.12E-07	11.7645	7.72E-07	1.21E-06	1.12E-09
Ozone formation, Terrestrial ecosystems	kg NOx eq	0.007321	0.007281	0.000518	7.07469	0.00643	0.008472486	5.18E-06
Ozone formation, Human health	kg NOx eq	0.00693	0.006888	0.000501	7.230699	0.0060741	0.008044788	5.01E-06
Mineral resource scarcity	kg Cu eq	0.010476	0.00948	0.004604	43.95008	0.0047455	0.022272766	4.60E-05
Marine eutrophication	kg N eq	0.000611	0.000607	7.26E-05	11.88417	0.0004809	0.000763915	7.26E-07
Marine ecotoxicity	kg 1,4-DCB	0.209529	0.184711	0.11655	55.62483	0.0655024	0.49802572	0.0011655
Land use	m^2a crop eq	0.054749	0.051911	0.013642	24.91744	0.0375927	0.089178655	0.00013642
Ionizing radiation	kBq Co-60 eq	0.308056	0.173948	0.438192	142.2445	0.0383696	1.3852809	0.00438192
Human non-carcinogenic toxicity	kg 1,4-DCB	2.926817	2.609568	1.411839	48.23803	1.2488585	6.4826961	0.01411839
Human carcinogenic toxicity	kg 1,4-DCB	0.235151	0.199417	0.199106	84.67166	0.1000503	0.56122336	0.00199106
Global warming	kg CO_2 eq	4.355413	4.343792	0.267043	6.131292	3.8626057	4.917179	0.00267043
Freshwater eutrophication	kg P eq	0.001057	0.000956	0.00048	45.40998	0.0004759	0.002303043	4.80E-06
Freshwater ecotoxicity	kg 1,4-DCB	0.162253	0.142221	0.092847	57.22393	0.0480297	0.39466789	0.00092847
Fossil resource scarcity	kg oil eq	1.700244	1.696149	0.120874	7.109234	1.4721599	1.9505012	0.00120874
Fine particulate matter formation	kg PM2.5 eq	0.004281	0.004251	0.000335	7.837293	0.0037355	0.005045432	3.35E-06

The PLA LCIA is considered to incorporate its feedstock impact into the lactic acid production. Thus, similarly, the PLA production system's (Polylactide, granulate {GLO}| market for | Cut-off) ReCiPe Midpoint (H) LCIA is shown in **Table S7**, while the uncertainty analysis due to these variables is summarised in **Table S8.**Similar to the lactic acid production system, the PLA system also has the highest variations in water consumption. The GWP value varies between 2.7 and 3.5 kg CO_2e/kg PLA at 2.5% and 97.5% deviations, with the hotspot being the electricity use.

Next, we consider two business-as-usual waste management systems (plastic incineration with energy recovery and landfill) to compare their environmental incentives with our PLA recycling system (see Supporting Information for methods of LCIA generation). To make better comparisons among different impact categories, a normalisation method was used. The ReCiPe Midpoint (H) provides the normalisation factors, where global warming potential has a lower factor than most of the impact categories. The normalisation factor is the ratio between an impact in a category of a system and the total impact in a region in the category. Because the total GWP is very high globally (ReCiPe Midpoint (H)), its normalisation factor appears to be lower than other impact categories. Normalisation is only used to compare the impact characterisations of a system.[11]

Figure S3 shows their normalised impact comparisons. There are some savings or avoided impacts (shown by negative impacts) in some categories, such as fossil resource scarcity, for the waste plastic incineration with energy recovery avoiding equivalent natural gas for energy generation systems. Also, except for water consumption, human carcinogenic toxicity, terrestrial ecotoxicity, stratospheric ozone depletion and GWP, waste plastic incineration with

energy recovery avoiding equivalent natural gas for energy generation systems are better performing than waste plastic landfill systems.

Table S7. ReCiPe Midpoint (H) LCIA of 1 kg Polylactide, granulate {GLO}| market for | Cut-off (1,4-DCB is the 1,4-dichlorobenzene equivalent representing the toxicity categories).

Impact category	Unit	Total	Polylactide, granulate {GLO}| production | Cut-off, U	Chemical factory, organics {GLO}| market for | Cut-off, U	maize grain {BR}| market for maize grain | Cut-off, U	maize grain {RoW}| market for maize grain | Cut-off, U	maize grain {ZA}| market for maize grain | Cut-off, U	naphtha {BR}| market for naphtha | Cut-off, U	naphtha {CO}| market for naphtha | Cut-off, U	naphtha {IN}| market for naphtha | Cut-off, U	naphtha {PE}| market for naphtha | Cut-off, U	Naphtha {RER}| market for | Cut-off, U	Naphtha {RoW}| market for | Cut-off, U	naphtha {ZA}| market for naphtha | Cut-off, U	Natural gas, high pressure {GLO}| market group for | Cut-off, U	Electricity, low voltage {GLO}| market group for | Cut-off, U
Global warming	kg CO_2 eq	3.06E+00	0.00E+00	5.94E-02	1.76E-02	8.06E-01	1.72E-03	3.47E-05	1.45E-05	3.74E-04	7.19E-05	3.95E-04	1.83E-03	2.95E-06	1.49E-03	1.37E+00
Stratospheric ozone depletion	kg CFC11 eq	1.09E-05	0.00E+00	2.73E-08	2.69E-07	9.66E-06	2.40E-08	8.56E-11	2.94E-11	7.00E-10	1.77E-10	9.09E-10	4.09E-09	6.88E-12	1.24E-09	5.48E-07
Ionizing radiation	kBq Co-60 eq	1.90E-01	0.00E+00	6.37E-03	2.22E-04	1.49E-02	2.73E-05	3.19E-06	1.06E-06	2.51E-05	6.59E-06	3.73E-05	1.53E-04	2.56E-07	1.47E-05	1.64E-01
Ozone formation, Human health	kg NOx eq	7.63E-03	4.54E-04	1.92E-04	3.45E-05	3.14E-03	6.72E-06	1.88E-07	6.82E-08	1.86E-06	3.91E-07	2.02E-06	9.51E-06	1.53E-08	3.02E-06	2.97E-03
Fine particulate matter formation	kg PM2.5 eq	5.21E-03	0.00E+00	1.48E-04	6.35E-05	1.70E-03	3.35E-06	1.07E-07	3.84E-08	1.10E-06	2.20E-07	1.22E-06	5.68E-06	9.02E-09	1.79E-06	2.98E-03
Ozone formation, Terrestrial ecosystems	kg NOx eq	8.02E-03	7.31E-04	1.95E-04	3.52E-05	3.19E-03	6.84E-06	2.01E-07	7.24E-08	1.96E-06	4.17E-07	2.15E-06	1.01E-05	1.63E-08	3.36E-06	2.99E-03
Terrestrial acidification	kg SO_2 eq	1.31E-02	0.00E+00	2.88E-04	4.30E-04	6.93E-03	1.36E-05	3.10E-07	1.13E-07	3.27E-06	6.38E-07	3.60E-06	1.66E-05	2.64E-08	5.47E-06	4.55E-03
Freshwater eutrophication	kg P eq	1.17E-03	0.00E+00	6.18E-05	3.61E-06	2.82E-04	6.19E-07	3.40E-09	1.29E-09	3.54E-08	7.02E-09	4.07E-08	1.76E-07	3.25E-10	6.47E-08	6.70E-04
Marine eutrophication	kg N eq	1.33E-03	0.00E+00	3.02E-06	6.51E-05	8.33E-04	1.10E-06	3.21E-10	1.63E-10	4.37E-09	6.35E-10	3.95E-09	1.71E-08	2.79E-11	1.59E-08	4.57E-05
Terrestrial ecotoxicity	kg 1,4-DCB	3.81E+00	0.00E+00	5.60E-01	4.16E-02	2.11E+00	4.92E-03	6.26E-05	3.16E-05	1.45E-03	1.24E-04	9.42E-04	4.29E-03	4.84E-06	2.63E-04	9.88E-01
Freshwater ecotoxicity	kg 1,4-DCB	1.50E-01	0.00E+00	3.38E-02	8.01E-04	3.31E-02	8.35E-05	4.63E-07	2.24E-07	5.78E-06	9.60E-07	5.45E-06	2.45E-05	3.74E-08	1.19E-05	7.76E-02
Marine ecotoxicity	kg 1,4-DCB	1.82E-01	0.00E+00	4.27E-02	7.02E-04	3.49E-02	8.84E-05	8.02E-07	3.62E-07	9.64E-06	1.66E-06	9.36E-06	4.22E-05	6.45E-08	1.91E-05	9.77E-02
Human carcinogenic toxicity	kg 1,4-DCB	1.64E-01	0.00E+00	3.43E-02	8.22E-04	4.74E-02	1.25E-04	2.74E-06	9.47E-07	2.37E-05	5.69E-06	2.94E-05	1.34E-04	2.21E-07	6.85E-05	6.81E-02
Human non-carcinogenic toxicity	kg 1,4-DCB	2.24E+00	0.00E+00	4.54E-01	-6.63E-02	6.15E-01	2.29E-03	1.31E-05	6.95E-06	1.88E-04	2.70E-05	1.61E-04	7.24E-04	1.10E-06	3.07E-04	1.16E+00
Land use	m^2a crop eq	5.52E-01	0.00E+00	6.76E-03	3.84E-02	4.85E-01	7.15E-05	5.64E-07	1.91E-07	4.57E-06	1.16E-06	5.96E-06	2.68E-05	4.63E-08	4.50E-06	1.94E-02
Mineral resource scarcity	kg Cu eq	8.00E-03	0.00E+00	2.55E-03	9.12E-05	3.72E-03	1.02E-05	5.94E-08	3.89E-08	1.01E-06	1.23E-07	7.61E-07	3.43E-06	4.75E-09	1.43E-06	1.29E-03
Fossil resource scarcity	kg oil eq	8.12E-01	0.00E+00	1.42E-02	2.71E-03	1.52E-01	3.38E-04	1.16E-04	3.87E-05	9.08E-04	2.41E-04	1.22E-03	5.50E-03	9.28E-06	3.42E-03	3.42E-01
Water consumption	m^3	1.28E-01	3.20E-03	6.91E-04	2.04E-04	1.16E-01	7.17E-05	3.73E-07	1.54E-07	3.57E-06	8.81E-07	3.71E-07	1.32E-06	3.25E-08	1.27E-06	1.03E-02

Impact category	Heat, district or industrial, natural gas {GLO}| market group for | Cut-off, U	Heat, district or industrial, other than natural gas {GLO}| market group for | Cut-off, U	Hazardous waste, for incineration {CH}| market for hazardous waste, for incineration | Cut-off, U	Hazardous waste, for incineration {Europe without Switzerland} | market for hazardous waste, for incineration | Cut-off, U	Hazardous waste, for incineration {RoW}| market for hazardous waste, for incineration | Cut-off, U	Waste plastic, mixture {BR}| market for waste plastic, mixture | Cut-off, U	Waste plastic, mixture {CO}| market for waste plastic, mixture | Cut-off, U	Waste plastic, mixture {CY}| market for waste plastic, mixture | Cut-off, U	Waste plastic, mixture {IN}| market for waste plastic, mixture | Cut-off, U	Waste plastic, mixture {PE}| market for waste plastic, mixture | Cut-off, U	Waste plastic, mixture {RER}| market group for waste plastic, mixture | Cut-off, U	Waste plastic, mixture {RoW}| market for waste plastic, mixture | Cut-off, U	Waste plastic, mixture {ZA}| market for waste plastic, mixture | Cut-off, U	Wastewater from maize starch production {GLO}| market for | Cut-off, U	Waste plastic, mixture {RER}| market group for waste plastic, mixture | Cut-off, U	Waste plastic, mixture {RoW}| market for waste plastic, mixture | Cut-off, U	Waste plastic, mixture {ZA}| market for waste plastic, mixture | Cut-off, U	Wastewater from maize starch production {GLO}| market for | Cut-off, U
Global warming	7.45E-01	1.71E-02	1.30E-04	5.06E-03	1.06E-02	2.95E-05	2.17E-06	1.20E-07	2.51E-06	3.95E-07	1.35E-04	4.89E-04	7.70E-07	2.25E-02	1.92E-07	3.01E-07	1.06E-10	-2.72E-03
Stratospheric ozone depletion	2.50E-07	3.52E-09	4.17E-11	1.63E-09	3.41E-09	5.56E-12	5.12E-13	1.19E-14	1.13E-12	1.07E-13	5.58E-11	1.84E-10	1.43E-13	8.16E-08	1.02E-06	2.20E-06	1.32E-08	4.20E-03
Ionizing radiation	2.06E-03	8.28E-05	2.13E-06	8.47E-05	1.57E-04	2.56E-08	1.62E-09	5.63E-11	5.86E-10	2.65E-10	7.89E-08	1.41E-07	7.26E-10	1.73E-03	1.63E-08	2.94E-08	9.05E-11	6.14E-05
Ozone formation, Human health	6.73E-04	4.28E-05	9.32E-08	4.06E-06	8.77E-06	3.96E-08	3.81E-09	1.04E-10	7.67E-09	7.75E-10	5.18E-08	8.27E-07	1.18E-09	9.09E-05	2.00E-07	1.47E-06	7.42E-09	3.91E-04
Fine particulate matter formation	2.12E-04	3.74E-05	5.56E-08	2.31E-06	4.97E-06	3.64E-08	3.91E-09	8.79E-11	9.02E-09	8.25E-10	2.17E-08	1.60E-06	1.09E-09	4.86E-05	2.79E-04	3.05E-03	1.39E-05	1.86E-02
Ozone formation, Terrestrial ecosystems	7.04E-04	4.31E-05	9.47E-08	4.13E-06	8.91E-06	4.05E-08	3.90E-09	1.06E-10	7.84E-09	7.92E-10	5.23E-08	8.43E-07	1.21E-09	9.15E-05	2.44E-06	1.25E-05	2.84E-08	1.66E-03
Terrestrial acidification	6.35E-04	9.78E-05	1.21E-07	5.12E-06	1.09E-05	1.81E-08	1.70E-09	4.71E-11	3.24E-09	3.40E-10	2.50E-08	3.48E-07	5.47E-10	1.27E-04	1.72E-05	1.49E-04	7.52E-07	1.41E-03
Freshwater eutrophication	9.74E-06	4.76E-06	3.70E-08	1.43E-06	2.98E-06	1.11E-10	6.98E-12	2.05E-13	2.66E-12	1.15E-12	4.87E-10	9.01E-10	7.12E-12	1.30E-04	1.29E-05	1.10E-04	5.54E-07	1.10E-03
Marine eutrophication	1.51E-06	3.02E-07	2.13E-09	8.25E-08	1.72E-07	1.94E-07	1.28E-08	1.00E-09	3.97E-09	2.10E-09	7.80E-08	1.04E-06	5.09E-09	3.79E-04	9.26E-05	2.83E-03	1.02E-06	2.38E-02
Terrestrial ecotoxicity	4.92E-02	1.55E-02	7.38E-05	3.86E-03	8.25E-03	4.19E-05	3.59E-06	7.76E-08	5.67E-06	6.96E-07	9.26E-05	2.83E-03	1.02E-06	2.38E-02	7.80E-08	1.04E-06	5.09E-09	3.79E-04
Freshwater ecotoxicity	2.31E-03	2.26E-04	2.26E-06	8.91E-05	1.86E-04	1.86E-05	1.22E-06	9.59E-08	3.82E-07	2.25E-07	1.29E-05	1.10E-04	5.54E-07	1.10E-03	4.87E-10	9.01E-10	7.12E-12	1.30E-04
Marine ecotoxicity	3.42E-03	3.17E-04	3.06E-06	1.21E-04	2.53E-04	2.52E-05	1.65E-06	1.30E-07	5.16E-07	3.05E-07	1.72E-05	1.49E-04	7.52E-07	1.41E-03	2.50E-08	3.48E-07	5.47E-10	1.27E-04
Human carcinogenic toxicity	9.18E-03	4.61E-04	1.52E-05	5.88E-04	1.22E-03	6.80E-07	5.64E-08	2.31E-09	8.80E-08	1.41E-08	2.44E-06	1.25E-05	2.84E-08	1.66E-03	5.23E-08	8.43E-07	1.21E-09	9.15E-05
Human non-carcinogenic toxicity	3.85E-02	1.25E-02	5.22E-05	2.06E-03	4.33E-03	5.22E-04	3.44E-05	2.68E-06	1.11E-05	5.74E-06	2.79E-04	3.05E-03	1.39E-05	1.86E-02	2.17E-08	1.60E-06	1.09E-09	4.86E-05
Land use	9.46E-04	1.00E-03	5.50E-07	2.43E-05	5.52E-05	2.54E-07	1.76E-08	1.04E-09	6.85E-09	3.00E-09	2.00E-07	1.47E-06	7.42E-09	3.91E-04	5.18E-08	8.27E-07	1.18E-09	9.09E-05
Mineral resource scarcity	2.41E-04	3.13E-06	7.96E-08	3.63E-06	7.11E-06	2.80E-09	1.71E-10	5.80E-12	6.23E-11	2.79E-11	1.63E-08	2.94E-08	9.05E-11	6.14E-05	7.89E-08	1.41E-07	7.26E-10	1.73E-03
Fossil resource scarcity	2.81E-01	3.13E-03	1.31E-05	5.39E-04	1.13E-03	5.45E-07	3.46E-08	1.34E-09	1.20E-08	5.60E-09	1.02E-06	2.20E-06	1.32E-08	4.20E-03	5.58E-11	1.84E-10	1.43E-13	8.16E-08
Water consumption	8.09E-04	2.60E-05	4.71E-07	1.77E-05	3.69E-05	4.63E-09	1.59E-10	4.81E-12	6.02E-11	2.62E-11	1.92E-07	3.01E-07	1.06E-10	-2.72E-03	1.35E-04	4.89E-04	7.70E-07	2.25E-02

Table S8. Statistical analyses of ReCiPe Midpoint (H) categories of Polylactide, granulate {GLO}| market for | Cut-off due to deviations of the variables in Ecoinvent 3.10.

Impact category	Unit	Mean	Median	SD	CV	2.50%	97.50%	SEM
Water consumption	m^3	1.23E-01	1.60E-01	7.15E-01	5.83E+02	-1.43E+00	1.41E+00	7.15E-03
Terrestrial ecotoxicity	kg 1,4-DCB	3.81E+00	3.71E+00	6.86E-01	1.80E+01	2.82E+00	5.45E+00	6.86E-03
Terrestrial acidification	kg SO_2 eq	1.31E-02	1.31E-02	9.91E-04	7.55E+00	1.13E-02	1.52E-02	9.91E-06
Stratospheric ozone depletion	kg CFC11 eq	1.09E-05	1.08E-05	1.45E-06	1.33E+01	8.37E-06	1.40E-05	1.45E-08
Ozone formation, Terrestrial ecosystems	kg NOx eq	8.03E-03	7.99E-03	6.41E-04	7.99E+00	6.87E-03	9.38E-03	6.41E-06
Ozone formation, Human health	kg NOx eq	7.64E-03	7.60E-03	6.19E-04	8.11E+00	6.52E-03	8.95E-03	6.19E-06
Mineral resource scarcity	kg Cu eq	7.98E-03	7.47E-03	2.22E-03	2.78E+01	5.31E-03	1.37E-02	2.22E-05
Marine eutrophication	kg N eq	1.33E-03	1.32E-03	1.36E-04	1.02E+01	1.09E-03	1.62E-03	1.36E-06
Marine ecotoxicity	kg 1,4-DCB	1.81E-01	1.72E-01	6.31E-02	3.48E+01	9.32E-02	3.34E-01	6.31E-04
Land use	m^2a crop eq	5.52E-01	5.46E-01	7.49E-02	1.36E+01	4.24E-01	7.19E-01	7.49E-04
Ionizing radiation	kBq Co-60 eq	1.92E-01	1.10E-01	2.85E-01	1.49E+02	2.70E-02	8.40E-01	2.85E-03
Human non-carcinogenic toxicity	kg 1,4-DCB	2.22E+00	2.03E+00	9.59E-01	4.31E+01	1.08E+00	4.51E+00	9.59E-03
Human carcinogenic toxicity	kg 1,4-DCB	1.62E-01	1.39E-01	1.21E-01	7.46E+01	7.45E-02	3.75E-01	1.21E-03
Global warming	kg CO_2 eq	3.06E+00	3.05E+00	2.15E-01	7.03E+00	2.66E+00	3.51E+00	2.15E-03
Freshwater eutrophication	kg P eq	1.17E-03	1.08E-03	4.36E-04	3.73E+01	6.29E-04	2.23E-03	4.36E-06
Freshwater ecotoxicity	kg 1,4-DCB	1.49E-01	1.42E-01	5.03E-02	3.37E+01	7.76E-02	2.69E-01	5.03E-04
Fossil resource scarcity	kg oil eq	8.13E-01	8.10E-01	6.65E-02	8.17E+00	6.94E-01	9.54E-01	6.65E-04
Fine particulate matter formation	kg PM2.5 eq	5.22E-03	5.19E-03	4.32E-04	8.28E+00	4.45E-03	6.15E-03	4.32E-06

A new waste plastic incineration data was created based on “Waste plastic, mixture {RoW}| treatment of waste plastic, mixture, municipal incineration | Cut-off” updated with avoided natural gas (calorific value = 8.125 kWh/m^3) (Ecoinvent 3.10 life cycle inventory database: “Natural gas, high pressure {GLO}| market group for | Cut-off”). For the energy recovery in the waste plastic incineration, a calorific value of 30.79 MJ/kg waste plastics and 75% electricity generation efficiency are considered.[12]This new waste plastic incineration data and the life cycle inventory “Waste plastic, mixture {RoW}| treatment of waste plastic, mixture, sanitary landfill | Cut-off” were run to generate LCIA results which were normalised for comparisons (**Figure S3**).

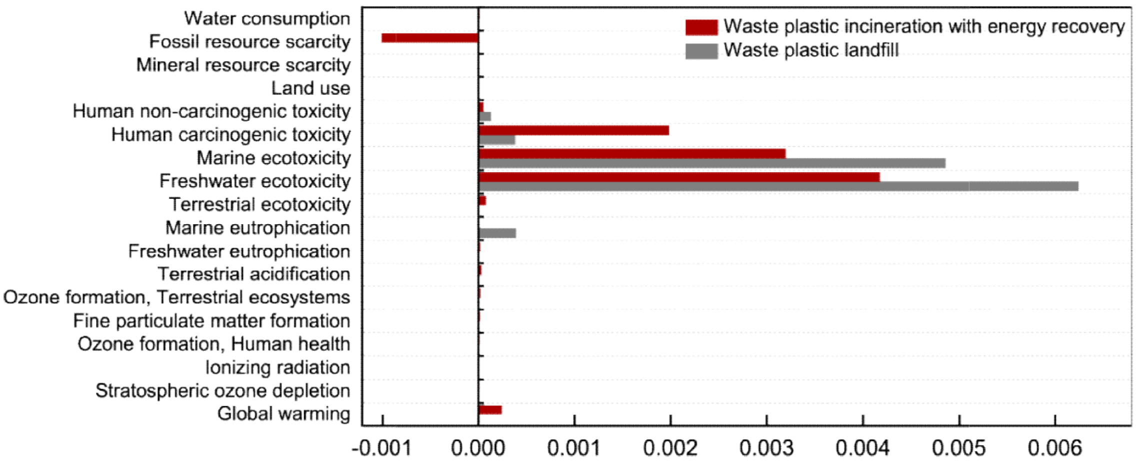

Figure S3. Normalised LCIA comparisons between waste plastic incineration with energy recovery avoiding equivalent natural gas and waste plastic landfill systems.

The mechanochemical PLA recycling into the LA production process at the laboratory consumes sodium hydroxide or potassium hydroxide and electricity, as shown in **Table S9**, requiring their lifecycle impact consideration through the Ecoinvent 3.10 life cycle inventory data assimilation in **Table S3**. **Table S9**shows the inventory flow data basis for the two scales considered for the new system, Scenario 1: optimal upscaled system and Scenario 2: laboratory system. The electricity use is estimated from measured data, similar to previous literature reports on calculating conversions between rotational and angular velocity; angular velocity and torque; and torque and energy.[13,14]The caustic soda or sodium hydroxide (NaOH) can be recovered to the rate of 95% or more and thus, only the makeup NaOH should comprise its consumption rate in the up-scaled process.

Table S9. Inventory data for electricity and material in PLA recycling into LA systems for Scenario 1: optimal upscaled system (top) and Scenario 2: laboratory system (bottom).

**Scenario 1: optimal upscaled system**	Inventory flow
Electricity (kWh/kg PLA)	0.58<br>Basis:<br>Ball milling: 850rpm for 10 mins and 10mm diameter<br>Ageing: at 60°C for 3 days<br>Calculations:

	Ball milling:<br>Angular velocity = $2\pi \times \frac{1}{60} \times 850rpm = 89\ radian/s$<br>Torque $= 0.5 \times 9.81 \times 1.6 \times 0.005 = 0.039$ Newton-m (where the proportionality constant that depends on the distribution of the load and the friction characteristics is 0.5; the total mass of the balls and materials is 1.6 kg)<br>Electrical energy use $= 0.89 \times 0.039 \times 10 \times 60 \times \frac{1}{10^3 \times 3.6} = 0.575$ kWh/kg<br>Ageing:<br>Electrical energy use $= \frac{1}{72} \times 43.8 \times (60 - 18) \times \frac{1}{10^3 \times 3.6} = 0.007$ kWh/kg (the glass transition temperature of amorphous PLA occurs at T=332.5 K with a change in heat capacity of 43.8 $J \cdot K^{-1} \cdot mol^{-1}$; the molar mass of PLA is 72; ambient temperature is 18°C)
Sodium hydroxide (kg/kg PLA)	0.039 (make-up); 1.073 (in-process recycled)
LA (kg/kg PLA)	LA product yield: 92.06%, LA molecular weight 90.08 g/mol, so LA yield:<br>1/72×92.06%×90.08 = 1.152 (kg/kg PLA)

**Scenario 2: laboratory system**	Inventory flow
Electricity (kWh/kg PLA)	1.704 (including RAM, ageing and electrodialysis)<br>Basis:<br>Resodyn acoustic mixer (RAM): The instrument is operating at 100G, 1A, 100V for 10 min. 13.927g PLA was treated in one batch, therefore the electricity consumption is:<br>1×100×(10/60)/(13.927/1000) = 1.197 kWh/kg<br>Ageing: at 60°C for 1 day<br>Electrical energy use $= \frac{1}{72} \times 43.8 \times (60 - 18) \times$

	$\frac{1}{10^3 \times 3.6}$ = 0.007 kWh/kg (the glass transition temperature of amorphous PLA occurs at T=332.5 K with a change in heat capacity of 43.8 J · K^{-1} · mol^{-1}; the molar mass of PLA is 72; ambient temperature is 18°C)<br>Electrodialysis: the specific energy consumption for LA is 0.036 kWh/mol, so the total energy for 1kg PLA is 0.036×(1000/72) = 0.5 kWh/kg PLA
potassium hydroxide or sodium hydroxide (kg/kg PLA)	0.7443 kg (KOH) or 0.532 kg (NaOH)<br>(the values above are based on the following recycling: with each mol of LA, one mole of KOH or NaOH is recycled as by-product from LA electrodialysis)
LA (kg/kg PLA)	LA product yield: 91.01%, LA molecular weight 90.08 g/mol, so LA yield:<br>1/72×91.01%×90.08 = 1.139 (kg/kg PLA)

Table S10. ReCiPe Midpoint (H) LCIA of the mechanochemical PLA recycling into the LA production system (Scenario 1: optimal upscaled system) (Basis: 1 kg PLA recycling). The overall impact column has been highlighted, while the other columns represent the dominance analysis.

**Impact category**	**Unit**	**Total**	**Sodium hydroxide, without water, in 50% solution state {GLO}| market for | Cut-off, U**	**Electricity, medium voltage {RER}| market group for | Cut-off, U**
Global warming	kg CO_2eq	2.90E-01	5.13E-02	2.38E-01
Stratospheric ozone depletion	kg CFC11 eq	1.67E-07	5.46E-08	1.12E-07

Ionizing radiation	kBq Co-60 eq	1.27E-01	5.61E-03	1.21E-01
Ozone formation, Human health	kg NOx eq	5.64E-04	1.35E-04	4.29E-04
Fine particulate matter formation	kg PM2.5 eq	4.64E-04	1.11E-04	3.53E-04
Ozone formation, Terrestrial ecosystems	kg NOx eq	5.69E-04	1.36E-04	4.33E-04
Terrestrial acidification	kg SO_2eq	1.06E-03	1.86E-04	8.78E-04
Freshwater eutrophication	kg P eq	2.59E-04	2.51E-05	2.34E-04
Marine eutrophication	kg N eq	1.92E-05	2.56E-06	1.66E-05
Terrestrial ecotoxicity	kg 1,4-DCB	2.68E-01	9.79E-02	1.70E-01
Freshwater ecotoxicity	kg 1,4-DCB	1.27E-02	2.85E-03	9.90E-03
Marine ecotoxicity	kg 1,4-DCB	1.69E-02	3.70E-03	1.32E-02
Human carcinogenic toxicity	kg 1,4-DCB	2.14E-02	4.23E-03	1.72E-02
Human non-carcinogenic toxicity	kg 1,4-DCB	3.58E-01	5.98E-02	2.99E-01
Land use	m^2a crop eq	6.84E-03	1.18E-03	5.67E-03
Mineral resource scarcity	kg Cu eq	4.27E-04	1.63E-04	2.64E-04
Fossil resource scarcity	kg oil eq	7.66E-02	1.28E-02	6.39E-02
Water consumption	m^3	5.37E-03	1.35E-03	4.02E-03

Table S11 ReCiPe Midpoint (H) LCIA of the mechanochemical PLA recycling into the LA production system (Basis: 1 kg LA production). The overall impact column has been highlighted, while the other columns represent the dominance analysis.

**Impact category**	**Unit**	**Total**	**Polylactide, granulate {GLO}| market for | Cut-off, U**	**Electricity, medium voltage {RER}| market group for | Cut-off, U**	**Sodium hydroxide, without water, in 50% solution state {GLO}| market**

					**for | Cut-off, U**
Global warming	kg CO_2eq	2.96E+00	2.71E+00	2.06E-01	4.44E-02
Stratospheric ozone depletion	kg CFC11 eq	9.58E-06	9.44E-06	9.72E-08	4.72E-08
Ionizing radiation	kBq Co-60 eq	2.75E-01	1.66E-01	1.05E-01	4.85E-03
Ozone formation, Human health	kg NOx eq	7.57E-03	7.08E-03	3.71E-04	1.16E-04
Fine particulate matter formation	kg PM2.5 eq	5.04E-03	4.64E-03	3.06E-04	9.56E-05
Ozone formation, Terrestrial ecosystems	kg NOx eq	7.92E-03	7.43E-03	3.75E-04	1.17E-04
Terrestrial acidification	kg SO_2eq	1.26E-02	1.17E-02	7.59E-04	1.61E-04
Freshwater eutrophication	kg P eq	1.24E-03	1.02E-03	2.02E-04	2.17E-05
Marine eutrophication	kg N eq	1.17E-03	1.15E-03	1.44E-05	2.22E-06
Terrestrial ecotoxicity	kg 1,4-DCB	4.54E+00	4.31E+00	1.47E-01	8.46E-02
Freshwater ecotoxicity	kg 1,4-DCB	1.42E-01	1.31E-01	8.56E-03	2.46E-03
Marine ecotoxicity	kg 1,4-DCB	1.74E-01	1.60E-01	1.14E-02	3.20E-03
Human carcinogenic toxicity	kg 1,4-DCB	1.67E-01	1.49E-01	1.49E-02	3.65E-03
Human non-carcinogenic toxicity	kg 1,4-DCB	2.29E+00	1.98E+00	2.58E-01	5.17E-02
Land use	m^2a crop eq	4.87E-01	4.81E-01	4.90E-03	1.02E-03
Mineral resource scarcity	kg Cu eq	7.44E-03	7.07E-03	2.29E-04	1.41E-04

Fossil resource scarcity	kg oil eq	7.91E-01	7.25E-01	5.52E-02	1.10E-02
Water consumption	m^3	1.16E-01	1.11E-01	3.47E-03	1.16E-03

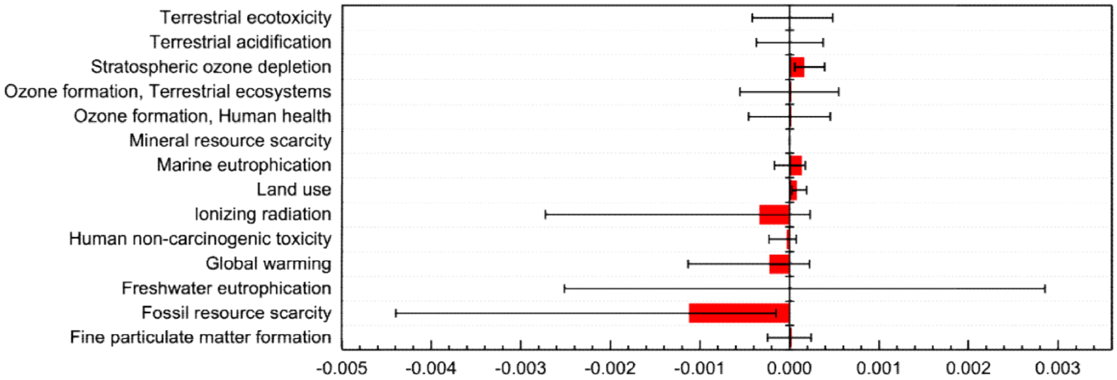

Figure S4. Normalised ReCiPe (M) (H)distributions for the new mechanochemical PLA recycling system run with renewable electricity. Amongst these impact categories, fossil resource scarcity and freshwater eutrophication are the most uncertain impact categories, while the other impact categories including GWP show certainties or robustness, and hence, confidence in results.

Reference

[1] J. A. Gerde, M. Montalbo-Lomboy, L. Yao, D. Grewell, T. Wang, *BioresourTechnol***2012**, *125*, 175.

[2] Q. Wang, G. Q. Chen, L. Lin, X. Li, S. E. Kentish, *Sep PurifTechnol***2021**, *279*, 119739.

[3] M. Atasoy, I. Owusu-Agyeman, E. Plaza, Z. Cetecioglu, *BioresourTechnol***2018**, *268*, 773.

[4] M. Courtat, P. J. Joyce, S. Sim, J. Sadhukhan, R. Murphy, *J Environ Manage***2023**, *336*, 117684.

[5] R. I. Muazu, J. Sadhukhan, S. Venkata Mohan, S. Gadkari, *Environ Sci (Camb)***2023**, *9*, 2487.

[6] J. Sadhukhan, *Renew Energy***2022**, *184*, 960.

[7] J. Sadhukhan, *Energies 2022, Vol. 15, Page 5522***2022**, *15*, 5522.

[8] J. Sadhukhan, S. Sen, T. M. S. Randriamahefasoa, S. Gadkari, *Digital Chemical Engineering* **2022**, *3*, 100026.

[9] S. Wang, F. Li, J. Sadhukhan, J. Xuan, X. Mao, L. Xing, X. Zhao, X. Wang, *J Clean Prod* **2024**, *434*, 139846.

[10] "ecoinvent v3.10 - ecoinvent," can be found under https://ecoinvent.org/ecoinvent-v3-10/, **n.d.**

[11] J. Sadhukhan, K. S. Ng, E. M. Hernandez, *Biorefineries and Chemical Processes: Design, Integration and Sustainability Analysis* **2014**, *9781119990864*, 1.

[12] J. Sadhukhan, E. Martinez-Hernandez, *BioresourTechnol* **2017**, *243*, 135.

[13] R. Borges, M. Brondi, A. M. Elias, C. S. Farinas, C. Ribeiro, *J Environ Manage* **2024**, *363*, 121254.

[14] E. Anglou, Y. Chang, A. Ganesan, S. Nair, C. Sievers, F. Boukouvala, *Computer Aided Chemical Engineering* **2023**, *52*, 2405.
